# Placenta Thickness Mediates the Association Between *AKIP1* Methylation in Maternal Peripheral Blood and Full-Term Small for Gestational Age Neonates

**DOI:** 10.3390/genes15121510

**Published:** 2024-11-25

**Authors:** Huimin Zhu, Min Wei, Xuemei Liu, Xiuxiu Li, Xuhua Liu, Weiqing Chen

**Affiliations:** 1 Department of Epidemiology, School of Public Health, Sun Yat-sen University, Guangzhou 510080, China; zhuhm9@mail2.sysu.edu.cn; 2 Department of Science and Education, Shenzhen Birth Cohort Study Center, Nanshan Maternity and Child Healthcare Hospital, Shenzhen 518067, China; weiminphd@126.com (M.W.); liuxm@szbcs.org (X.L.); lixiuxiu1103@163.com (X.L.); liuxh@szbcs.org (X.L.)

**Keywords:** AKIP1, DNA methylation, placental thickness, SGA, mediation effect

## Abstract

Background/Objectives: A-kinase-interacting protein 1 (AKIP1) has been discovered to be a pivotal signaling adaptor in the regulation of human labor and associated with preterm birth, but its effect on fetal growth was still unclear. Meanwhile, the regulation role of DNA methylation (DNAm) on placental and fetal development has been demonstrated. Therefore, we aimed to investigate the association of *AKIP1* DNAm in maternal peripheral blood with placental development and full-term small for gestational age (FT-SGA) neonates, and to explore whether placenta mediate the association between *AKIP1* DNAm and FT-SGA; Methods: This study was a case–control study including 84 FT-SGAs and 84 FT-AGAs derived from the Shenzhen Birth Cohort Study. The DNA methylation analysis of CpG in the target region of the *AKIP1* gene was measured by the Sequenom MassARRAY EpiTYPER approach. Multiple-variable logistic and linear regression analyses were used to estimate the association between the DNAm of three validated CpG sites in the *AKIP1* gene, placental thickness, and FT-SGA. Mediation analysis was used to examine the mediation effect of placental development on the association between the DNAm of *AKIP1* and FT-SGA. Results: For every increment in standard deviation in the DNAm of CpG4 (cg00061907) at *AKIP1*, the risk of FT-SGA elevated by 2.01-fold (aOR = 2.01, 95%CI = 1.39~3.01), and the thickness of the placenta significantly decreased by a 0.19 standard deviation (β = −0.19, 95%CI = −0.32~ −0.06). Placental thickness mediated the 22.96% of the effect of the DNAm of CpG4 at *AKIP1* on the risk of FT-SGA with statistical significance. Conclusions: The findings in the present study suggested the mediating effect of placental thickness on the association of the DNAm of *AKIP1* in maternal peripheral blood and the risk of FT-SGA, providing new evidence for the mechanism of maternal epigenetics in placental and fetal development.

## 1. Introduction

Small for gestational age (SGA) neonates, which refers to newborns with a birthweight (BW) less than the 10th percentile for gestational age, are at a higher risk of a range of short-term or long-term adverse health effects such as neonatal mortality and morbidity, poor neurodevelopment, and cardiovascular disease in later life [1,2]. In 2020, 23.4 million (17.4%) liveborn babies were SGA, accounting for 14.7% of attributed mortality globally [3]. It is widely agreed that SGAs are composed of constitutionally small and growth-restricted fetuses [4]. To distinguish the growth-restricted SGAs from constitutionally small but heathy SGAs, we focused on SGAs with a gestational age from 37 to 42 weeks, which are referred to full-term small for gestational age neonates in this study.

The placenta is a transient but irreplaceable organ that plays important roles in fetal growth during pregnancy through placental transportation, maternal–placental circulation, and the modulation of maternal endocrine and metabolism [5]. In a prospective cohort study, Chantale et al. found that reduced placenta thickness in the first trimester was found to be associated with SGA [6]. Placental thickness represents the extent of arborization of the villous capillary bed, where maternal fetal exchange actually happens [7]. However, the molecular mechanism of placental development is still not fully elucidated.

A-kinase-interacting protein 1 (AKIP1) has been discovered as an important signaling adaptor in the regulation of human labor and has a pivotal significance for preterm birth through interacting with protein kinase A [8]. Additionally, AKIP1 was also reported as an activator of nuclear factor-κB (NF-κB), which had been shown to be a key mediator of placentation and the remodeling of uterine spiral arteries during pregnancy [8]. The failure of uterine spiral arteries is associated with reduced villous development and maternal–fetal exchange [9]. On the other hand, the role of DNA methylation on fetal growth and placental development has been demonstrated in recent studies [10,11,12,13]. Therefore, the association between the DNAm of *AKIP1* with both placental development and fetal growth is worth further investigation.

Based on these findings, we made the hypothesis that changes in *AKIP1* DNAm could contribute to the failure of uterine spiral arteries’ remodeling and implantation of the placenta, inducing poor fetal growth. Given the growing evidence indicating that the DNAm of maternal and fetal tissues were involved in fetal growth, and the presence of fetal or placental DNA was observed in maternal peripheral blood [14,15], we aimed to investigate the association of *AKIP1* DNAm in maternal peripheral blood with placental development and full-term SGA, and to explore whether placenta mediate the association between *AKIP1* DNAm and full-term SGA.

## 2. Materials and Methods

### 2.1. Study Population

The Shenzhen Birth Cohort Study (SZBCS, NCT03830879) is a population-based prospective cohort study conducted in Shenzhen Nanshan Maternity and Child Healthcare Hospital, which was designed to explore the environmental and genetic influence during pregnancy on fetal growth, birth outcome, and childhood development. Informed consent was obtained from all participants in this cohort study. Participants were recruited if they were before 19 weeks of gestation and planned to receive maternal and child healthcare in this hospital. During pregnancy, participants attended 3 follow-up visits, in which social demography information, medical history, lifestyle and environmental information, clinical examinations, and biological samples were collected. After delivery, the birth outcome was recorded and childhood follow-up initialed, including growth measurements, biological sample collection, and questionnaires about child nurturing. The study was conducted in accordance with the Declaration of Helsinki and approved by the Ethics Committee of Nanshan Maternity and the Child Healthcare Hospital of Shenzhen (NSFYEC-KY-2020031) and Sun Yat-sen University (2018-054).

This study was a case–control study derived from SZBCS. Mother–infant pairs with a gestational age of 37 to 42 week and BW < 10th percentile according to the population-based sex-specific reference were recognized as cases, while those with a gestational age of 37 to 42 week and BW ranging from the 10th to 90th percentiles, which is full-term-appropriate for gestational age (FT-AGA), were defined as the control [16]. The individuals in the case and control were matched by pre-pregnancy BMI (the same), parity (the same), and gestational age (±7 days). The participants with multiple pregnancies, maternal kidney disease, anemia, infection disease, or diabetes mellitus (gestational diabetes mellitus), hypertension (gestational hypertension), fetal hereditary disease, or malformation were excluded in this study.

### 2.2. Data Collection

Participants were required to complete a questionnaire about social demography information and medical history and reproductive history at the first follow-up visit after recruitment, and the questionnaire data were entered into database. Birth outcomes including gestational age at the time of delivery, sex, birth length, BW, and head circumference of the neonates were obtained from the Hospital Information System. The gestational age was determined by the scan of crown–rump length (CRL) at 11–13 weeks of gestation.

### 2.3. Measurement

Placental characteristics were measured by a trained midwife immediately after delivery. The weight of the placenta was determined with a weighing machine in a scale interval of 0.01 g. The maximum and minimum axis of the placenta, which are used to calculate the placental surface area, was measured using standard non-elastic tape in a scale interval of 1 mm. The thickness of the placenta was measured once for the central zone, twice for the middle zone and peripheral using a long needle, and the mean of five measurements was recorded as the thickness of the placenta [17].

### 2.4. Sample Collection and DNA Extraction

Maternal peripheral blood samples of 5 mL were collected in the first trimester of pregnancy using EDTA tubes and stored at −80 °C on the same day. DNA of samples was extracted with a QIAamp DNA Mini Kit (QIAGEN, Dusseldorf, Germany) in accordance with manufacturer’s instructions, and the DNA concentration and purity was assessed by NanoDrop 2000 (Thermo Scientific™, San Jose, CA, USA) and DNA gel electrophoresis (Major science). DNA samples with a total DNA of 20 μg, molecular size more than 20 kb, and A260/A280 ranging from 1.7 to 2.1 were qualified for the following DNA methylation measurement.

### 2.5. DNA Methylation Analysis Using Sequenom MassARRAY Platform

Prior to the DNA methylation analysis of CpG in the target region, an epigenome-wide dataset for another 8 pairs of case and control matched by pre-pregnancy BMI, parity, and gestational age was generated using Infinium Methylation EPICv2.0 (935 k) Beadchip (Illumina, San Diego, CA, USA) in accordance with the manufacturer’s protocol. The quality control details before the identification of differentially methylated CpG positions (DMPs) are displayed in Supplementary File S1. The methylation levels of qualified CpG sites were presented as a β value, and those with |Δβ| ≥ 0.2 and adjusted *p* value < 0.05 were identified as DMPs. Two of the DMPs (cg00061907, cg15696082) were annotated to the AKIP1 gene (Supplementary File S2: Table S1). 

The DNA methylation analysis of CpG in the target region of the AKIP1 gene was measured by the Sequenom MassARRAY EpiTYPER approach. The primers for base pairs 947-1381 of AKIP1 were designed with Agena EpiDesigner (http://www.epidesigner.com, accessed on 11 March 2024). The target region was composed of 13 CpG sites, among which CpG3 and CpG8 were not examined because no signal was detectable. The methylation profiles of CpG13 and CpG14 were estimated based on the average level of two sites, as the molecular weight of the two sites overlapped. In total, 10 CpG sites, which includes 1 multiple CpG sites, were examined in this study. The details of the target region are shown in Supplementary File S2: Table S2. The forward and backward primers for the target region were aggaagagagTTTAAAATGTTGGGATTATAGGTGG and cagtaatacgactcactatagggagaaggctCCCTAATCCTCAAAAAACCTAAAAA.

Before measurement, bisulfite treatment and PCR amplification for each sample was performed using an EZ DNA Methylation-Gold Kit (Zymo Research, Orange, CA, USA) and PCR Accessory Set (SEQUENOM, San Diego, CA, USA) in accordance with the manufacturer’s protocol. Quantitative methylation analysis was then conducted with the Sequenom MassARRAY platform, which utilizes a combination of matrix-assisted laser desorption/ionization time-of-flight (MALDI-TOF) mass spectrometry and RNA base-specific cleavage, to detect the AKIP1 methylation profile quantitatively. The quantitative results for each CpG site, or an aggregate of multiple CpG sites, was generated by EpiTYPER software 1.0 (SEQUENOM, San Diego, USA). The DNA methylation level is presented as a β value and ranges from 0 (unmethylated) to 1 (fully methylated).

Several approaches were applied to control the quality of the DNA methylation analysis. Analyses for cases and controls were performed within the same batch to reduce the batch effect. A total of 5% of samples in the case group and control group were randomly retested to confirm the repeatability of results. An external unmethylated segment was induced during bisulfite treatment to verify the efficiency of bisulfite conversion. For the detected samples, those with more than half of the CpG sites having a poor readout (the β value was 0, 1, or NA) were considered unacceptable and excluded from further analysis. For the detected CpG sites, those with a poor readout in more 10% of samples, and those with an average β-value less than 0.05 or more than 0.95, were eliminated. Three CpG sites were included in the final analysis. For the flowchart of quality control, see Supplementary File S2: Figure S1.

### 2.6. Statistical Analyses

To describe the data, values were presented as means and standard deviation or the median and interquartile range, depending on whether normally distributed or not, for continuous variables and as a number (%) for categorical variables. The distribution of continuous variables and categorical variables was compared using Student’s *t* test and chi-squared test, respectively.

Several multivariable logistic regression analyses were performed to examine the association of FT-SGA with the DNAm level of *AKIP1* and placental characteristics. According to the relevant studies, covariates including maternal age, pre-pregnancy BMI, marriage status, monthly income, education level, employment status, smoking and alcohol intake before pregnancy, and reproductive history were adjusted in the multivariable logistic regression. A sensitivity analysis, in which pre-pregnancy smoking was not included in the multivariable logistic regression model, was performed to examine the effect of pre-pregnancy smoking on the association of the DNAm level of *AKIP1* and FT-SGA. Another sensitivity analysis was conducted by evaluating the association of the DNAm level of *AKIP1* and FT-SGA among participants, excluding those exposed to prenatal alcohol, to test the influence of alcohol use on the association.

Multivariable linear regression analyses were applied to test the association of the DNAm of *AKIP1* and placental characteristics, adjusting for the covariates mentioned above. To ensure that coefficients were comparable across different models, the DNAm of *AKIP1* and placental characteristics were converted to standard scores using the formula z = (x − μ)/σ. Subsequently, mediation analysis was performed to explore the mediating role of placental characteristics on the association of the DNAm of *AKIP1* and FT-SGA. According to Kenny and Sobel, the mediation is presented when the following conditions were met: (1) the independent variable (the DNAm of *AKIP1* in this study) is significantly associated with dependent variable (FT-SGA in this study); (2) the independent variable (the DNAm of *AKIP1*) is significantly associated with the mediating variable (placental characteristics); (3) the mediating variable (placental characteristics) is significantly related to the dependent variable (FT-SGA); and (4) the association between the independent variable and dependent variable (FT-SGA) get weaker or insignificant when the mediating variable is additionally adjusted [18]. The covariates mentioned above were also controlled in the models of mediation analysis. The bootstrapping procedure of PROCESS macro was applied for the test of the mediation effect, which would be considered to be insignificant if the 95% confidence interval contained zero [19]. The proportion of mediation was estimated based on the method of VanderWeele, using the formula $\frac{{OR}_{DE}\times ({OR}_{IE}-1)}{{OR}_{DE}\times {OR}_{IE}-1}$ for continuous mediating variables and binary dependent variables [20].

All the statistical analyses were performed using R (version 4.2.2, R Foundation for Statistical Computing, Vienna, Austria). To reduce the Type I error, a Bonferroni corrected α was applied and a *p*-value < 0.017 (two-sided) was considered to be statistically significant in this study.

## 3. Results

### 3.1. Population Characteristics

This study included 84 pairs of qualified participants, and the characteristics of the study participants are shown in Table 1. Overall, the two groups were comparable in maternal characteristics, including pre-pregnancy BMI, marriage status, monthly income, education level, employment status, and reproductive history, including parity and method of conception. For placental characteristics, the participants in the FT-SGA group had significantly smaller and lighter placenta than the FT-AGA group. For infant’s characteristics, the birthweight, length, and head circumference of FT-SGA infants was significantly smaller than FT-AGA infants, but the sex and gestational weeks for the two groups were comparable.

### 3.2. Association of DNAm of AKIP1 and the Risk of FT-SGA

**Figure 1:**
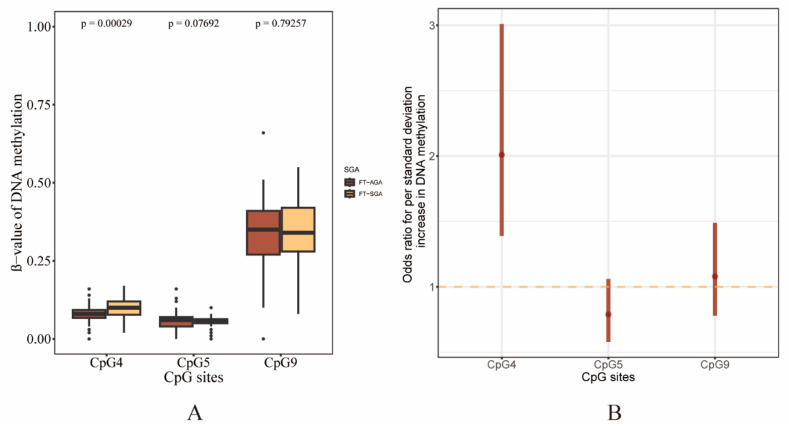
Differences of DNA methylation between groups. β value of DNA methylation distribution across CpGs grouped by FT-AGA and FT-SGA (**A**). Comparison of the association between DNA methylation levels and FT-SGA across CpGs (**B**).

As shown in Figure 1A, the mean β-value of CpG4 of participants in the FT-SGA group was significantly higher than the FT-AGA group. There was no statistically significant difference in the mean β-value of CpG5 and CpG9 between the FT-SGA and FT-AGA groups. The correlation coefficient between CpG4, CpG5, and CpG9 in Supplementary File S2: Figure S2 showed that CpG9 was correlated with both CpG4 and CpG5; therefore, Bonferroni correction for the three independent tests was applied in this study. 

In multivariable logistic regression analyses adjusted for potential covariates, when comparing the FT-SGA to the FT-AGA group, the DNAm of CpG4 at AKIP1 was related to a higher risk of SGA with an aOR of 2.01 (95%CI = 1.39–3.01) (Figure 1B, Table 2), showing that for every increment in standard deviation in the DNAm of CpG4 at AKIP1, the risk of FT-SGA elevated by 2.01-fold. The results of two sensitivity analyses demonstrated that the association was both robust in the model without adjustment for pre-pregnancy smoking and among the participants without pre-pregnancy alcohol intake (Supplementary File S2: Tables S3 and S4).

### 3.3. The Relationship of Placental Characteristics with the DNAm of AKIP1 and the Risk of FT-SGA

The results in multivariable linear regression analyses showed that the DNAm of CpG4 in AKIP1 was significantly related to placental thickness (β = −0.19, 95%CI = [−0.32, −0.06], *p* = 0.004) and placental weight (β = −0.20, 95%CI = [−0.36, −0.05], *p* = 0.011). Meanwhile, there was no statistical significance for the correction between the DNAm of CpG4 and placental area (β = −0.19, 95%CI = [−0.36, −0.03], *p* = 0.023) (Table 3).

As presented in Table 2, the thickness of placenta was significantly and negatively correlated with the risk of SGA, with an adjusted OR of 0.45 (95%CI = 1.39–3.01), which means a per standard deviation increase in placental thickness was associated with a 0.55-fold reduction in the risk of SGA. Additionally, as shown in Table 3, a decreased risk of SGA was associated with a higher placental area (aOR = 0.40, 95%CI = 0.23–0.62), as well as placental weight (aOR = 0.28, 95%CI = 0.14–0.47), with statistical significance.

### 3.4. Mediation Analysis

**Figure 2:**
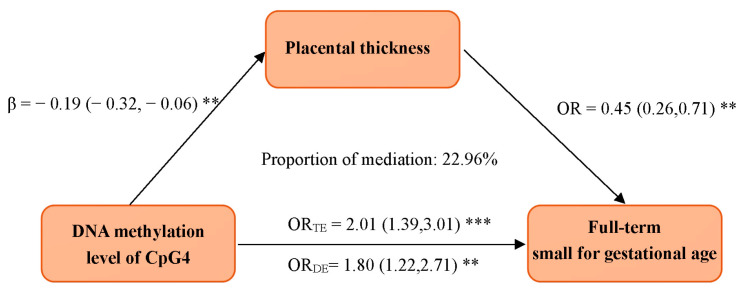
The mediation effect of placental thickness on the association of the DNA methylation of CpG4 in *AKIP1* and FT-SGA. Models were adjusted for maternal age, education status, marriage status, monthly income, employment, pre-pregnancy body mass index (BMI), conception method, and parity. **: *p* < 0.01; ***: *p* < 0.001. OR_TE_: odds ratio for total effect. OR_DE_: odds ratio for direct effect.

Mediation analyses for the mediating roles of placental thickness and placental weight on the association of the DNAm of CpG4 in AKIP1 and FT-SGA were performed. The effect of the association between the DNAm of CpG4 in AKIP1 and the risk of FT-SGA decreased and was still significant (aOR = 1.80, 95%CI = 1.22–2.71) when placental thickness was introduced into the model, compared to the model without adjustment for placental thickness (Figure 2). Further bias-corrected bootstrap 95% CIs for placental thickness ranged from 0.0012 to 0.0509, indicating the significant mediation effect of placental thickness, and the estimated proportion of mediation was 22.96%.

The association between the DNAm of CpG4 in AKIP1 and the risk of FT-SGA also get weaker and remain significant when placental weight (aOR = 1.92, 95%CI = 1.27–3.00) is additionally adjusted (Supplementary File S2: Figure S3). However, the bootstrapping test indicated that no significant mediation effect of placental weight (95%CI = −0.0026~0.0575) on the relationship between the DNAm of CpG4 in AKIP1 and the risk of FT-SGA was observed. 

## 4. Discussion

In this study, we found that the methylation level of CpG4 (cg00061907) in the *AKIP1* gene was higher in the maternal peripheral blood collected at the first trimester from the FT-SGA group when comparing to the FT-AGA group. A higher methylation level of CpG4 (cg00061907) in the *AKIP1* gene was associated with a decreased thickness of placenta and elevated risk of SGA. A further mediation analysis found that placental thickness mediates the association between CpG4 methylation in *AKIP1* and FT-SGA.

Preview studies have explored the DNAm level of placental tissue or cord blood and its regulation on fetal growth. In placental tissues, a retrospective study found the average DNAm level of *Rtl1* promoter differed between control, SGA, and severe SGA groups [10]. Another study collected placental tissues from IUGRs and AGAs and revealed that the DNAm level of *p66Shc* promoter decreased in IUGR placenta [21]. Diaz and colleagues found that in both placenta and cord blood from a SGA group that the DNAm levels of the *ATG2B*, *NKX6.1*, and *SLC13A5* genes were hypermethylated in two types of sample [22]. However, a limited investigation examined the DNAm level in the maternal peripheral blood of mothers who delivered SGA neonates. In fact, emerging evidence demonstrated that the DNAm of maternal and fetal tissues was involved in fetal growth [14]. Lo et al. revealed the presence of fetal DNA in maternal peripheral blood, while another study identified that 10% of cell-free DNA in maternal peripheral blood originates from the placenta [15,23]. These DNA derived from fetus and placenta could reflect epigenetic information, including DNA methylation [23]. In a prospective study including 163 participants, Gascoigne et al. found an association between epigenetic clock aging in maternal peripheral blood and preterm birth [24]. Yu et al. constructed an early prediction model for preeclampsia based on the different DNAm profiles in maternal peripheral blood between the case group and healthy controls [25]. Similarly, the results of our study found different DNAm levels of the *AKIP1* gene in maternal peripheral blood collected in the first trimester between SGA and AGA groups, also providing evidence for prenatal biomarkers and an early prediction of fetal growth.

The association between the DNAm level of the *AKIP1* gene and FT-SGA was discovered in this study. The risk of FT-SGA elevated by 2.01-fold for every increment in standard deviation in the DNAm of CpG4 at *AKIP1*. Investigations concerning AKIP1 and adverse birth outcomes were sparse. Yulia et al. collected myometrial biopsies from pregnant women and found that the mRNA and protein levels of AKIP1 would not increase until the onset of labor and were raised in a different phenotype of preterm labor. Further experiments in vitro demonstrated that the interaction of AKIP1 and PKA has a pivotal role in preterm and term birth during labor through mediating the regulation of cAMP and the expression of Cyclo-oxygenase-2, which is critical to the onset of human labor [8]. However, no further studies have been performed to investigate the association of AKIP1 and fetal growth. Therefore, the finding in this study still demands further validation through examination regarding the difference in the DNAm, mRNA, and protein levels of AKIP1 in placental tissues or other samples collected from AGA and SGA groups.

The results of this study also demonstrated the relationship between the DNAm level of the *AKIP1* gene and placental characteristics. In previous studies, the *AKIP1* gene and the protein it encoded were reported as novel biomarkers and as involved in the progression and prognosis of patients with cancers, including glioma, multiple myeloma, and thyroid carcinoma [26,27,28]. Furthermore, several research studies have uncovered the role of AKIP1 in the activation and regulation of NF-κB. Gao and colleagues have revealed the interaction of AKIP1 and the NF-κB p65 subunit and its enhancement of the expression of NF-κB-mediated genes [29]. The translocation rate of the NF-κB p65 subunit was also found to be regulated by the interaction of AKIP1 and PKA, according to the results of experiments in vitro [30]. More importantly, NF-κB acts as a key mediator of placentation and the remodeling of uterine spiral arteries. Armistead et al. reported that the level of MMP-2 and MMP-9, which facilitate implantation and trophoblast invasion through its critical role in extra-cellular matrix degradation and extra-villous cytotrophoblast invasion, was upregulated by NF-κB [31]. NF-κB was also reported to regulate macrophage polarization into the M2 phenotype, which is indispensable in inflammation limiting in the uterus and the process of spiral artery remodeling [32,33]. In this study, we found that a per standard deviation increase in the DNAm level of cg00061907, which is located in transcription start site TS1500, was associated with a 0.19- and 0.20-fold of standard deviation reduction in placental thickness and weight and was hypermethylated in maternal peripheral blood of participants with an SGA neonate. Several studies had reported that the hypermethylation of TS1500 in a specific gene was inversely associated with the amount of protein expression [34,35]. Therefore, the hypermethylation of TS1500 in AKIP1 might result in the reduced expression of the protein it encodes, leading to the reduced activation of NF-κB, which may impair cytotrophoblast invasion and spiral artery remodeling. Though further studies on the downstream functional role of different DNAm levels of the *AKIP1* gene are still needed, evidence from these studies might explain the potential mechanism underlying the association between the DNAm level of the *AKIP1* gene and placental characteristics.

The association between placental characteristics and birthweight has been illustrated. The characteristics of the placenta such as disc shape, diameter, and weight were also reported to be related to birthweight [36]. A population-based case–control study found that the reduction of placental thickness was independently associated with a lower birthweight [37]. In a prospective cohort study, reduced placenta thickness in the first trimester was found to be associated with SGA [6]. Consistently, the correlation between SGA and the characteristics of the placenta, including surface area, thickness, and weight, was also observed in this study. Additionally, it has been discovered that the placenta mediates maternal environmental or genetics factors that cause lower birthweight. For instance, the placenta was found to mediate the association between birthweight and prenatal environmental exposure, such as to Thallium, second-hand smoke [38,39], and maternal lifestyle factors such as anxiety and exercise [39,40]. For genetics factors, a study of the birth cohort revealed the mediating role of the placenta in the relationship between maternal hypertension polygenetic scores and the offspring birthweight [41]. Tian et al. also found the placental surface area to be a mediator for the effect of the DNAm level of *FGFR2* on the mother side of the placenta on full-term low birthweight [42]. The results of our study showed that the mediation effect of placental thickness and weight is presented as according to Kenny and Sobel. Therefore, the mediation effect of placental thickness and weight was tested using the bootstrapping procedure of PROCESS macro. The results demonstrated that 22.96% of the effect of the DNAm of CpG4 in *AKIP1* on the risk of SGA was significantly mediated by placental thickness. The potential mechanism is still unclear, but considering that placenta thickness represents the arborization of the villous capillary bed, the reduced villous development caused by *AKIP1* DNAm and NF-κB may explain the results.

To the best of our knowledge, this was the first study to elucidate the association of the DNAm of *AKIP1* in maternal peripheral blood and the risk of FT-SGA and the mediating role of placental thickness on this association. However, certain limitations, as follows, should also be addressed. Firstly, the study’s statistical power was limited owing to the small sample size, potentially impeding the observation of a robust association with greater variability or small effect size, though a Bonferroni corrected α has been applied to enhance the statistical power. For instance, we failed to observe a statistically significant mediation effect of placental weight, which may be due to the fact that its small-size effects are not detectable within the current sample size. Therefore, the results of this study still require further replication in a larger sample size. Secondly, we performed a DNA methylation analysis on the peripheral blood without adjustment for the effect of cell type in the testing sample. The possible variance owing to cell heterogeneity between case and control and its potential effects on our study results cannot be ignored. Thirdly, though a significant relationship of *AKIP1* DNAm and FT-SGA was observed, the results still need to be validated in peripheral blood collected from other stages of pregnancy and placental tissue obtained during delivery. Fourthly, the mRNA and protein level of AKIP1 were not measured to support the downstream functional role of different DNAm levels of the *AKIP1* gene in the present study. Though DNAm is recognized to be involved in the regulation of gene expression, and a significant difference between SGAs and AGAs was observed in this study, its contribution to the significance of *AKIP1* expression may be relatively small. Thus, examinations for the expression of the *AKIP1* gene and further animal experiments should be conducted in future studies. Fifthly, previous studies have found variations in DNAm among populations of different ethnicities, habitats, or living environments [43,44]. On the other hand, similar to most observational epidemiological studies, the findings of present study, which derived from one single maternal and children healthcare hospital, can only be representative for the population in Southern China. Therefore, the generalization of the results in the present study to other regions or ethnicities should be cautious. Multi-center studies that involve more regions and ethnicities are necessary for the validation of our results in future investigations.

## 5. Conclusions

The findings in the present study suggested the mediating effect of placental thickness on the association of the DNAm of *AKIP1* in maternal peripheral blood and the risk of FT-SGA, providing new evidence for the mechanism of maternal epigenetics in placental and fetal development.

## Figures and Tables

**Table 1:** Maternal, placental, and infant characteristics of participants stratified by FT-AGA and FT-SGA.

Characteristic	Overall (*N* = 168)	FT-AGA (*N* = 84)	FT-SGA (*N* = 84)	*p*-Value
Mothers				
Age (year), mean (SD)	30.27 (4.9)	31.24 (5.2)	29.30 (4.4)	0.010
Pre-pregnancy BMI, N (%)				>0.999
normal	108 (64.3)	54 (64.3)	54 (64.3)	
underweight	46 (27.4)	23 (27.4)	23 (27.4)	
overweight	14 (8.3)	7 (8.3)	7 (8.3)	
Marriage status, married, N (%)	164 (98%)	83 (99%)	81 (96%)	0.620
Monthly income, <5000/month, N (%)	22 (13%)	12 (14%)	10 (12%)	0.572
Education, high school or lower, N (%)	17 (10.1)	7 (8.3)	10 (11.9)	0.636
Employment status, employed, N (%)	154 (92%)	79 (94%)	75 (89%)	0.403
Pre-pregnancy smoking, N (%)	4 (2.4%)	2 (2.4%)	2 (2.4%)	>0.999
Pre-pregnancy alcohol intake, N (%)	15 (8.9%)	5 (6.0%)	10 (12%)	0.279
Parity, primiparity, N (%)	122 (73%)	61 (73%)	61 (73%)	>0.999
Conception method, spontaneous conception, N (%)	154 (92%)	76 (90%)	78 (93%)	0.781
Placenta				
Maximum axis (cm), mean (SD)	19.84 (1.3)	20.22 (1.2)	19.45 (1.3)	<0.001
Minimum axis (cm), mean (SD)	18.31 (1.3)	18.62 (1.5)	18.00 (1.0)	0.002
Thickness (cm), mean (SD)	2.56 (0.2)	2.63 (0.2)	2.49 (0.3)	<0.001
Area (cm^2^), mean (SD)	286.05 (36.1)	296.66 (39.4)	275.43 (29.0)	<0.001
Weight (gram), mean (SD)	509.34 (57.2)	528.96 (61.6)	489.71 (44.8)	<0.001
Infants				
Sex, female, N (%)	68 (40%)	46 (55%)	54 (64%)	0.271
Gestational weeks (w), mean (SD)	39.17 (1.2)	39.13 (1.3)	39.21 (1.1)	0.641
Birthweight (gram), mean (SD)	2954.80 (354.5)	3204.52 (279.6)	2705.07 (220.2)	<0.001
Birth length (cm), mean (SD)	48.98 (1.7)	49.89 (1.4)	48.07 (1.4)	<0.001
Head circumference (cm), mean (SD)	32.64 (1.2)	33.18 (1.0)	32.10 (1.1)	<0.001

Abbreviations: SD, standard deviation; BMI, body mass index.

**Table 2:** Association of validated CpG sites’ DNA methylation level and placental characteristics with the risk of FT-SGA.

	Mean (SD)		Odds Ratio for Per Standard Deviation Increments (95% CI) ^a^	*p* Value
	FT-AGA	FT-SGA		
AKIP1				
CpG4	0.08 (0.03)	0.10 (0.03)	2.01 (1.39, 3.01)	<0.001
CpG5	0.06 (0.03)	0.05 (0.02)	0.79 (0.58, 1.06)	0.122
CpG9	0.34 (0.13)	0.35 (0.10)	1.08 (0.78, 1.49)	0.662
Placental area	296.66 (39.44)	275.43 (28.99)	0.40 (0.23, 0.62)	<0.001
Placental thickness	2.63 (0.21)	2.49 (0.25)	0.45 (0.26, 0.71)	0.002
Placental weight	528.96 (61.61)	489.71 (44.83)	0.28 (0.14, 0.47)	<0.001

^a^ Models were adjusted for maternal age, education status, marriage status, monthly income, employment, pre-pregnancy body mass index (BMI), pre-pregnancy smoking, pre-pregnancy alcohol intake, conception method, and parity.

**Table 3:** Relationship between validated CpG sites’ DNA methylation level and placental characteristics.

AKIP1 CpG Sites	Adjusted Coefficient (95%CI) ^a^		
	Placental Area	Placental Thickness	Placental Weight
CpG4	−0.19 (−0.36, −0.03) *	−0.19 (−0.32, −0.06) **	−0.20 (−0.36, −0.05) *
CpG5	0.30 (0.17, 0.43) ***	−0.02 (−0.13, 0.09)	0.29 (0.16, 0.42) ***
CpG9	0.22 (0.07, 0.37) ***	−0.04 (0.16, 0.09)	0.20 (0.05, 0.34) **

^a^ Models were adjusted for maternal age, education status, marriage status, monthly income, employment, pre-pregnancy body mass index (BMI), conception method, and parity. *: *p* < 0.05; **: *p* < 0.01; ***: *p* < 0.001.

## Data Availability

The data supporting the findings of this study are available on request from the corresponding author. The data are not publicly available due to privacy or ethical restrictions.

## Supplementary Materials

The following supporting information can be downloaded at: https://www.mdpi.com/article/10.3390/genes15121510/s1. Table S1. Characteristics of differentially methylated CpG sites annotated to AKIP1 between FT-SGA and FT-AGA. Table S2. PCR primers for AKIP1 gene region. Table S3. Sensitivity analyses on the association of validated CpG sites’ DNA methylation level with the risk of FT-SGA. Table S4. Sensitivity analyses on the associations between the validated CpG sites’ DNA methylation level and the risk of FT-SGA after excluding the subjects with pre-pregnancy alcohol intake. Figure S1. Quality control flow chart. Figure S2. The correlation of DNA methylation across all the valid CpG sites invalid samples. Figure S3. The mediation effect of placental weight on the association of DNA methylation of CpG4 in AKIP1 and FT-SGA. Refs. [45,46,47,48,49,50] are cited in Supplementary Materials.
