# Supplementary material for: Placenta Thickness Mediates the Association Between AKIP1 Methylation in Maternal Peripheral Blood and Full-Term Small for Gestational Age Neonates
Source: Genes (Basel). 2024 Nov 25;15(12):1510. doi: 10.3390/genes15121510 (PMC11675766; doi:10.3390/genes15121510)
Supplement: Supplementary file 1 [file genes-15-01510-s001.zip › Supplementary File S1.pdf]

Additional file 1

The Infinium Methylation EPICv2.0 analysis

The Infinium Methylation EPICv2.0 analysis of 8 pairs of case and control were conducted using ChAMP 2.23.1 in R 4.2.1 and Bioconductor 3.16. Methylation level is represented by the average β-value = M/ (M+ U+ ε), where M and U represent the intesity of methylated and unmethylated allele, and ranged from 0 (unmethylated) to 1 (full methylated). In probes quality control, we excluded 2491 probes with poor detection (detection p-value <0.01 in one or more samples), 24173 probes with <3 beads in at least 5% of samples per probe, 3641 non-CpG probes, 31366 SNP-associated probes, 0 multi-hit probes, and 21443 probes located in chromosome X and Y [1,2]. A total of 853941 probeswere included for subsequent analysis. We applied the β-mixture quantile intra sample normalization procedure (BMIQ), which provide better nomalization for EPIC array data, to adjust type-I and type-II bias [3]. Then, Combat normalization method and RefbaseEWAS method were used for batch effect correction and adjust the heterogeneity correction [166,167,168]. All CpGs were annotated using EPICanno.ilm10b4.hg19, and the differential methylated CpGs position (DMPs) that further adjust for maternal age, pre-pregnancy BMI and gestational age were generated based on the champ.DMP function. The adjusted p values were calculated using the Benjamini–Hochberg method.

References

[1] Fortin J P, Triche T J, Jr., Hansen K D. Preprocessing, normalization and integration of the Illumina HumanMethylationEPIC array with minfi [J]. Bioinformatics, 2017, 33(4): 558-60.

[2] Zhou W, Laird P W, Shen H. Comprehensive characterization, annotation and innovative use of Infinium DNA methylation BeadChip probes [J]. Nucleic Acids Res, 2017, 45(4): e22.

[3] Teschendorff A E, Marabita F, Lechner M, et al. A beta-mixture quantile normalization method for correcting probe design bias in Illumina Infinium 450 k DNA methylation data [J]. Bioinformatics, 2013, 29(2): 189-96.

[4] Teschendorff A E, Menon U, Gentry-Maharaj A, et al. An epigenetic signature in peripheral blood predicts active ovarian cancer [J]. PLoS One, 2009, 4(12): e8274.

[5] Johnson W E, Li C, Rabinovic A. Adjusting batch effects in microarray expression data using empirical Bayes methods [J]. Biostatistics, 2007, 8(1): 118-27.

[6] Houseman E A, Accomando W P, Koestler D C, et al. DNA methylation arrays as surrogate measures of cell mixture distribution [J]. BMC Bioinformatics, 2012, 13: 86.
