# Supplementary material for: Placenta Thickness Mediates the Association Between AKIP1 Methylation in Maternal Peripheral Blood and Full-Term Small for Gestational Age Neonates
Source: Genes (Basel). 2024 Nov 25;15(12):1510. doi: 10.3390/genes15121510 (PMC11675766; doi:10.3390/genes15121510)
Supplement: Supplementary file 1 [file genes-15-01510-s001.zip › Supplementary File S2.pdf]

Additional File 2

Table S1. Characteristics of differentially methylated CpG sites annotated to *AKIP1* between FT-SGA and FT-AGA

Site	Mean		Δβ	Adjusted *p* value [a]	CHR	MAPINFO	Relation to Island	Gene annotation	UCSC RefGene Group
	FT-AGA	FT-SGA							
cg00061907	0.32	0.57	0.25	0.002	11	8909938	Shore	AKIP1	TS1500
cg15696082	0.28	0.49	0.21	0.002	11	8909996	Shore	AKIP1	TS1500

[a]p value adjusted with the Benjamini–Hochberg method

CHR： chromosome.

Table S2. PCR primers for *AKIP1* gene region

Chromosomal Region [a]	CHR11: 8908938 8910995
Target sequence (3'→5)	ggtgattaggccaaactgtgtggctgccaaggtggcctttggagaagggaacagctCGggattgaggCGagaCGgaggCGgggc<br>tgtctgcagatgttcctggccccCGgggcagggggCGcctgagaagttgtagggtctctcataatccctcagCGcagctctctctcccc<br>tccagtgggaagtaCGgattcacatatttttggagacctgttctcttggggtctgactccatcttttaggaagactgcaccatgtttaagtaagga<br>actgtgaatgcttacttaaaccatgtttaagtaaggacctgtcactccactcCGctggttatttggtaggagatacagcccaggctctcactgcta<br>attagtgactcagcCGcaccacccagCGcaaagcaaagCGcCGatgcttaggaaggtccactttgaggga
Primers	Forward: aggaagagag TTTAAAATGTTGGGATTATAGGTGG
	Reverse: cagtaatacgactcactatagggagaaggct CCCTAATCCTCAAAAAACCTAAAAA
Amplicon length	435
Number of CpG sites	13

[a] UCSC GRCh37/hg19

Table S3. Sensitivity analyses on the association of validated CpG sites' DNA methylation level with the risk of FT-SGA

	Mean (SD)		Odds ratio for per standard deviation increments (95% CI) [a]	*P* value
	FT-AGA	FT-SGA		
AKIP1				
CpG4	0.08 (0.03)	0.10 (0.03)	2.01 (1.38,3.01)	<0.001
CpG5	0.06 (0.03)	0.05 (0.02)	0.79 (0.58,1.06)	0.123
CpG9	0.34 (0.13)	0.35 (0.10)	1.07 (0.78,1.49)	0.665

[a]Models were adjusted for maternal age, education status, marriage status, monthly income, employment, prepregnancy body mass index (BMI), pre-pregnancy alcohol intake, conception method and parity.

Table S4. Sensitivity analyses on the associations between the validated CpG sites' DNA methylation level and the risk of FT-SGA after excluding the subjects with pre-pregnancy

alcohol intake.

	Mean (SD)		Odds ratio for per standard deviation increments (95% CI) [a]	*P* value
	FT-AGA N=79	FT-SGA N=74		
AKIP1				
CpG4	0.08 (0.03)	0.09 (0.03)	2.06 (1.40,3.31)	<0.001
CpG5	0.06 (0.04)	0.05 (0.02)	0.79 (0.58,1.07)	0.137
CpG9	0.34 (0.13)	0.34 (0.10)	1.06 (0.76,1.49)	0.727

[a]Models were adjusted for maternal age, education status, marriage status, monthly income, employment, prepregnancy body mass index (BMI), pre-pregnancy alcohol intake, conception method and parity.

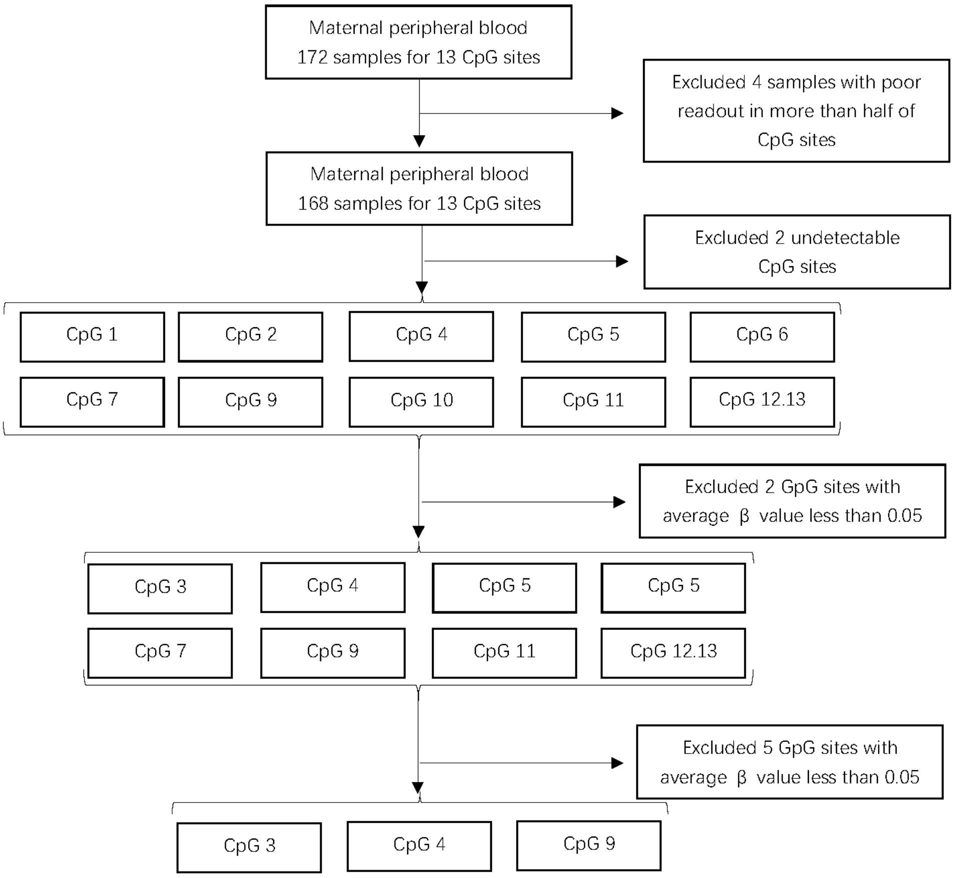

Figure S1. Quality control flow chart

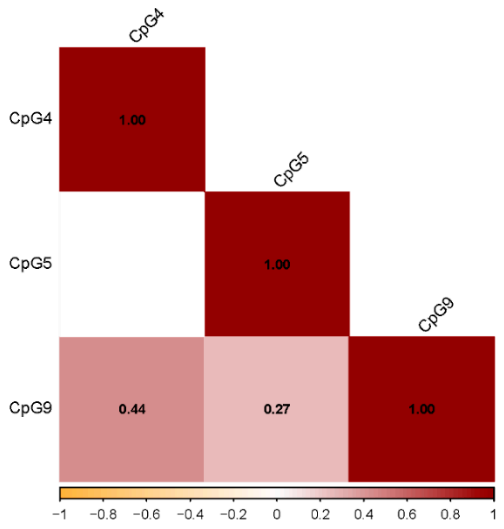

Figure S2. The correlation of DNA methylation across all the valid CpG sites invalid samples.

The correlation coefficients with a white background are not statistically significant ($P > 0.05$).

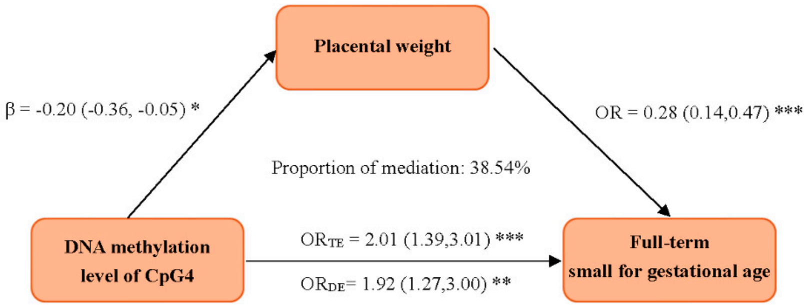

FigureS3 The mediation effect of placental weight on the association of DNA methylation of CpG4 in *AKIP1* and FT-SGA

Models were adjusted for maternal age, education status, marriage status, monthly income, employment, pre-pregnancy body mass index (BMI), conception method and parity. *: $p < 0.05$; **: $p < 0.01$; ***: $p < 0.001$. OR_{TE}: Odds ratio for total effect. OR_{DE}: Odds ratio for direct effect.
